# Supplementary material for: Malignant cerebral infarction after ChAdOx1 nCov-19 vaccination: a catastrophic variant of vaccine-induced immune thrombotic thrombocytopenia
Source: Nat Commun. 2021 Aug 2;12:4663. doi: 10.1038/s41467-021-25010-x (PMC8329262; doi:10.1038/s41467-021-25010-x)
Supplement: Supplementary file 1 — Supplementary Information [file 41467_2021_25010_MOESM1_ESM.pdf]

Supplementary Information for

Malignant cerebral infarction, systemic venous thrombosis and thrombocytopenia after ChAdOx1 nCov vaccination: a possible catastrophic variant of vaccine induced thrombotic thrombocytopenia

M. De Michele*, M. Iacobucci, A. Chistolini, E. Nicolini, F. Pulcinelli, B. Cerbelli, E. Merenda, O.G. Schiavo, E. Sbardella, I. Berto, L. Petraglia, N. Caracciolo, M. Chiara, S. Truglia, D. Toni

* Corresponding author. Email: M.DeMichele@policlinicoumberto1.it

The PDF file includes:

1. Supplementary Figure 1 page 2
2. Supplementary Figure 2 page 3
3. Supplementary Table 1 page 4

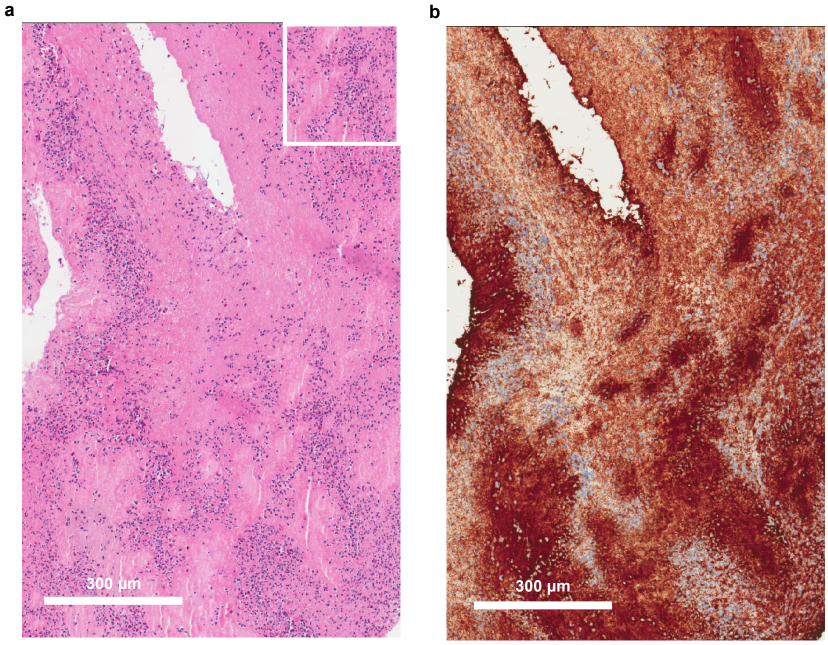

Supplementary Figure 1. Microscopic view of the retrieved clot from patient 1, during first endovascular thrombectomy.

a. Platelet-rich thrombus massively infiltrated by neutrophils. Morphologic features suggestive of Neutrophil DNA Extracellular Traps are present (insert). **b.** Immunohistochemistry for CD61 highlights the abundant presence of platelets. Immunohistochemical staining was performed three times using appropriate positive and negative controls with comparable results.

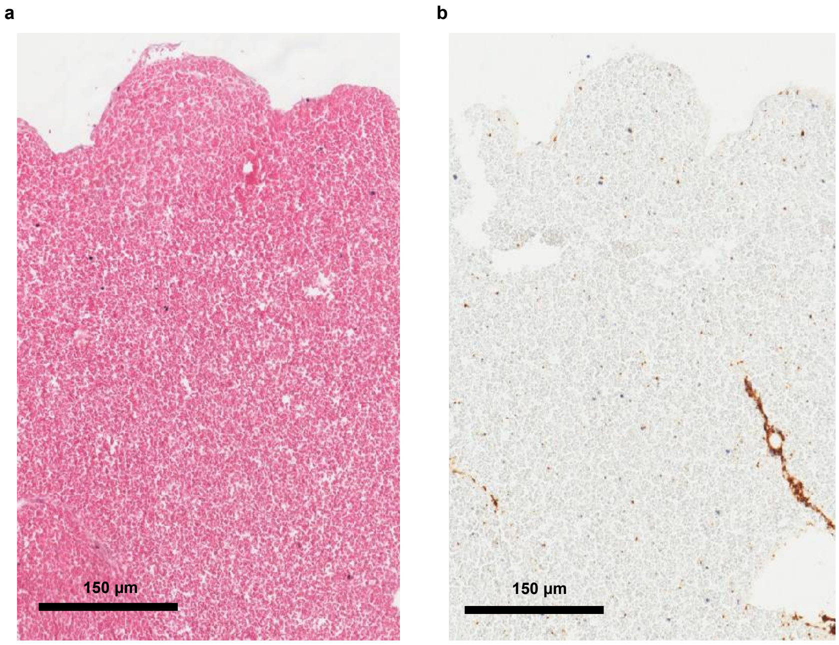

Supplementary Figure 2. Microscopic view of the retrieved clot from patient 1, during second endovascular thrombectomy.

a. Red blood cell-rich thrombus with scarce neutrophils. **b.** Immunohistochemistry for CD61 highlights very few platelets. Immunohistochemical staining was performed three times using appropriate positive and negative controls with comparable results.

Supplementary Table 1. Blood examination parameters at baseline, 12 hours, 24 hours and 48 hours in patient 1 and patient 2.

		PATIENT 1				PATIENT 2			
**Value**	**Reference value**	**T0**	**12 hours**	**24 hours**	**48 hours**	**T0**	**12 hours**	**24 hours**	**48 hours**
C-reactive protein (mg/dL)	0 - 0.5	12.13	7.79	NA	4.61	2.05	3.66	7.06	9.90
RBC (x10^6/µL)	3.5 - 5.1	4.45	3.8	3.55	3.54	4.41	4.18	4.11	3.46
Hemogobin (g/dL)	12 - 16	13.9	12.1	10.9	11.4	14.1	13.3	12.9	10.8
Hematocrit (%)	32 - 47	39.7	33.8	30.8	31.3	40.9	37.1	36.9	31.5
WBC (x10^3/µL)	4 - 10	6.08	6.98	9.59	7.01	8.8	6.96	9.59	6.06
Neutrophils (x10^3/µL)	2.2 - 6.6	2.76	6.16	8.08	5.72	7.2	6.18	8.68	5.23
Lymphocytes (x10^3/µL)	1 - 3.2	2.78	0.62	1.09	0.77	1	0.44	0.47	0.59
Monocytes (x10^3/µL)	0.2 - 1	0.28	0.11	0.33	0.36	0.6	0.19	0.35	0.21
Eosinophils (x10^3/µL)	0 - 0.8	0.12	0.03	0.01	0.06	0	0.1	0.02	0.01
Basophils (x10^3/µL)	0 - 1.5	0.02	0	0.01	0.01	0	0.01	0.01	0.01
Neutrophils (%)	40 - 74	45.4	88.3	84.3	81.6	82	88.8	90.5	86.3
Lymphocytes (%)	19 - 48	45.8	8.9	11.4	11	10.9	6.3	4.9	9.7
Monocytes (%)	3.4 - 11	4.6	1.6	3.4	5.1	6.3	2.8	3.7	3.4
Eosinophils (%)	0 - 7	1.9	0.5	0.1	0.8	0.6	1.4	0.2	0.1
Basophils (%)	0 - 2	0.4	0	0.1	0.1	0.2	0.2	0.1	0.1
Platelets count (x10^3/µL)	150 - 450	44	78	54	23	133	97	95	66
Reticulocyte index (%)	0.5 - 2.5	NA	NA	NA	2.8	NA	NA	NA	NA
Reticulocyte count (x10)	31 - 82	NA	NA	NA	96	NA	NA	NA	NA
Aspartate aminotransferase (UI/L)	8 - 38	55	33	NA	26	12.6	12	13	10
Alanine aminotransferase (UI/L)	12 - 41	83	62	NA	36	7.3	7	7	7
γGlutamyltransferase (UI/L)	5 - 36	110	92	NA	103	15.4	9	12	15
Lactate dehydrogenase (UI/L)	135 - 225	212	193	NA	256	NA	196	189	138
**Coagulation tests**									
		**PATIENT 1**				**PATIENT 2**			
**Value**	**Reference value**	**T0**	**12 hours**	**24 hours**	**48 hours**	**T0**	**12 hours**	**24 hours**	**48 hours**
PT INR	0.8 - 1.2	1.16	1.19	1.22	1.17	NA	1.04	1.16	1.18
PTT Ratio	0.8 - 1.2	0.83	0.84	0.86	0.83	0.99	0.85	1.17	0.94
Fibrinogen (mg/dL)	200 - 400	405	341	262	250	NA	385	336	322
D-DIMER (µg/L)	0 - 550	NA	NA	4318	>4318	5441	NA	31646	NA
Antithrombin III (%)	80 - 120	NA	NA	NA	96	NA	NA	84	NA
Factor XIII (%)	64 - 140	NA	NA	35.6	NA	NA	NA	NA	NA

Factor VIII (%)	58 - 130	NA	NA	143.7	NA	NA	NA	154	NA
C3 (mg/dL)	90 - 180	NA	NA	139	NA	NA	NA	NA	NA
C4 (mg/dL)	10 - 40	NA	NA	28.8	NA	NA	NA	NA	NA
VWF: RiCof (%)	41-124	NA	NA	342	NA	NA	NA	146	NA
VWF: Antigen (%)	41-130	NA	NA	145	NA	NA	NA	154	NA
**COVID 19 Testing**									
		**PATIENT 1**				**PATIENT 2**			
**Value**	**Reference value**	**T0**	**12 hours**	**24 hours**	**48 hours**	**T0**	**12 hours**	**24 hours**	**48 hours**
SARS-CoV-2 E Gene		ND	-	-		ND	-	-	
SARS-CoV-2 N Gene		ND	-	-		ND	-	-	
SARS-CoV-2 RdRp Gene		ND	-	-		ND	-	-	
SARS-CoV-2 RdRp/N Gene		ND	-	-		ND	-	-	
SARS-CoV-2 S Gene		ND	-	-		ND	-	-	
Ab Anti-Sars-CoV-2 (AU/ml)	positive >15	-	.	>400	-	-	-	<3.8	-

T0= baseline; NA= Not assessed; ND= Not detected; RBC= Red blood cells; WBC= White blood cells; RiCof= Ristocetin cofactor.

Laboratory data from case 1 and 2 have been reported for the first 48 hours. Severe thrombocytopenia and D-Dimer increase are evident in both patients.

From day 6, patient 1 showed progressively increase of liver enzymes peaking on day 17: aspartate aminotransferase (AST) at 487 UI/L, alanine aminotransferase (ALT) at 640 UI/L and γ-glutamyltransferase (GGT) at 163 UI/L (with the upper limits of normal range at 38 UI/L, 41 UI/L and 36 UI/L, respectively). Liver dysfunction was secondary to the portal vein thrombosis.
