# Supplementary material for: Malignant cerebral infarction after ChAdOx1 nCov-19 vaccination: a catastrophic variant of vaccine-induced immune thrombotic thrombocytopenia
Source: Nat Commun. 2021 Aug 2;12:4663. doi: 10.1038/s41467-021-25010-x (PMC8329262; doi:10.1038/s41467-021-25010-x)
Supplement: Supplementary file 3 — Reporting Summary [file 41467_2021_25010_MOESM3_ESM.pdf]

nature portfolio

Corresponding author(s): Manuela De Michele

Last updated by author(s): Jul 8, 2021

Reporting Summary

Nature Portfolio wishes to improve the reproducibility of the work that we publish. This form provides structure for consistency and transparency in reporting. For further information on Nature Portfolio policies, see our Editorial Policies and the Editorial Policy Checklist.

Statistics

For all statistical analyses, confirm that the following items are present in the figure legend, table legend, main text, or Methods section.

n/a	Confirmed	
☒	☐	The exact sample size (*n*) for each experimental group/condition, given as a discrete number and unit of measurement
☒	☐	A statement on whether measurements were taken from distinct samples or whether the same sample was measured repeatedly
☒	☐	The statistical test(s) used AND whether they are one- or two-sided<br>*Only common tests should be described solely by name; describe more complex techniques in the Methods section.*
☒	☐	A description of all covariates tested
☒	☐	A description of any assumptions or corrections, such as tests of normality and adjustment for multiple comparisons
☒	☐	A full description of the statistical parameters including central tendency (e.g. means) or other basic estimates (e.g. regression coefficient) AND variation (e.g. standard deviation) or associated estimates of uncertainty (e.g. confidence intervals)
☒	☐	For null hypothesis testing, the test statistic (e.g. *F*, *t*, *r*) with confidence intervals, effect sizes, degrees of freedom and *P* value noted<br>*Give P values as exact values whenever suitable.*
☒	☐	For Bayesian analysis, information on the choice of priors and Markov chain Monte Carlo settings
☒	☐	For hierarchical and complex designs, identification of the appropriate level for tests and full reporting of outcomes
☒	☐	Estimates of effect sizes (e.g. Cohen's *d*, Pearson's *r*), indicating how they were calculated

Our web collection on statistics for biologists contains articles on many of the points above.

Software and code

Policy information about availability of computer code

Data collection	No software was used for Data collection
Data analysis	No software was used for Data analysis

For manuscripts utilizing custom algorithms or software that are central to the research but not yet described in published literature, software must be made available to editors and reviewers. We strongly encourage code deposition in a community repository (e.g. GitHub). See the Nature Portfolio guidelines for submitting code & software for further information.

Data

Policy information about availability of data

All manuscripts must include a data availability statement. This statement should provide the following information, where applicable:

- Accession codes, unique identifiers, or web links for publicly available datasets
- A description of any restrictions on data availability
- For clinical datasets or third party data, please ensure that the statement adheres to our policy

Data of this report are available from the corresponding author upon reasonable request

Field-specific reporting

Please select the one below that is the best fit for your research. If you are not sure, read the appropriate sections before making your selection.

☒ Life sciences ☐ Behavioural & social sciences ☐ Ecological, evolutionary & environmental sciences

For a reference copy of the document with all sections, see nature.com/documents/nr-reporting-summary-flat.pdf

Life sciences study design

All studies must disclose on these points even when the disclosure is negative.

Sample size	No sample size calculation was perfomed as this study consists of two case reports
Data exclusions	No Data were excluded
Replication	Not applicable to this study
Randomization	Randomization is not applicable to this study, since it consists of two case reports
Blinding	authors who performed laboratory and histological analysis were blinded on clinical Data of patients

Reporting for specific materials, systems and methods

We require information from authors about some types of materials, experimental systems and methods used in many studies. Here, indicate whether each material, system or method listed is relevant to your study. If you are not sure if a list item applies to your research, read the appropriate section before selecting a response.

Materials & experimental systems

n/a	Involved in the study	
☐	☒	Antibodies
☒	☐	Eukaryotic cell lines
☒	☐	Palaeontology and archaeology
☒	☐	Animals and other organisms
☐	☒	Human research participants
☒	☐	Clinical data
☒	☐	Dual use research of concern

Methods

n/a	Involved in the study	
☒	☐	ChIP-seq
☒	☐	Flow cytometry
☐	☒	MRI-based neuroimaging

Antibodies

Antibodies used	Antibodies to platelet factor 4 (PF4)/polyanion IgG/IgA/IgM Immucor, Lifecodes, Waukesha, WI
Validation	LIFECODES PF4 Enhanced for in vitro diagnostic use [package insert], Waukesha, WI; Immucor GTI Diagnostics, Inc., 2013<br>LIFECODES PF4 IgG for in vitro diagnostic use [package insert], Waukesha, WI; Immucor GTI Diagnostics, Inc., 2013

Human research participants

Policy information about studies involving human research participants

Population characteristics	In the study we have described two females between 50 and 60 years of age, with acute ischemic stroke diagnosis and without past medical history relevant for the current diagnosis.
Recruitment	Healthy volunteers have been randomly recruited as healthy platelets and serum donors according to Italian regulation
Ethics oversight	Institutional review board at Sapienza University of Rome

Note that full information on the approval of the study protocol must also be provided in the manuscript.

Magnetic resonance imaging

Experimental design

Design type	N/A
Design specifications	N/A
Behavioral performance measures	N/A

Acquisition

Imaging type(s)	structural, diffusion, perfusion
Field strength	1.5 Tesla
Sequence & imaging parameters	1) Diffusion-Weighted Imaging (DWI) with b=0/1000s/mm2, 5 mm thick axial slices with corresponding apparent diffusion coefficient (ADC) mapping; 2) Fluid-Attenuated Inversion Recovery (FLAIR); 3) T1 sequences 4) T2 sequences 5) Grandient echo (GRE) sequences; 6) 3d Time-of-Flight (3D TOF) MR angiography (MRA); 7) Dynamic susceptibility contrast (DSC) MR perfusion-weighted imaging (PWI), and 8) T1 volumetric Interpolated Breath-hold Examination (VIBE) post injection of gadolinium contrast agent (on saggital plane)
Area of acquisition	whole brain scan

Diffusion MRI ☒ Used ☐ Not used

Parameters: large number of directions (>6), b value=0/1000s/mm, multi shell

Preprocessing

Preprocessing software	N/A
Normalization	N/A
Normalization template	N/A
Noise and artifact removal	N/A
Volume censoring	N/A

Statistical modeling & inference

Model type and settings	N/A
Effect(s) tested	N/A

Specify type of analysis: ☒ Whole brain ☐ ROI-based ☐ Both

Statistic type for inference (See Eklund et al. 2016)	N/A
Correction	N/A

Models & analysis

n/a	Involved in the study
☒	☐ Functional and/or effective connectivity
☒	☐ Graph analysis
☒	☐ Multivariate modeling or predictive analysis
